# Implications of perivascular spaces in amyotrophic lateral sclerosis: clinical significance and structural correlation

**DOI:** 10.1093/braincomms/fcaf448

**Published:** 2025-11-12

**Authors:** Sung-Ju Hsueh, Hsueh Wen Hsueh, Ya-Fang Chen, Ta-Fu Chen, Li-Kai Tsai, Ming-Chang Chiang, Sung-Tsang Hsieh, Wen-Chau Wu, Chi-Chao Chao

**Affiliations:** Department of Neurology, National Taiwan University Hospital Bei-hu Branch, Taipei 108206, Taiwan; Department of Neurology, National Taiwan University Hospital, Taipei 100225, Taiwan; Department of Neurology, National Taiwan University Hospital Yunlin Branch, Douliu City, Yunlin County 640203, Taiwan; Department of Neurology, National Taiwan University Hospital, Taipei 100225, Taiwan; Department of Neurology, National Taiwan University Hospital Hsinchu Branch, Zhubei City, Hsinchu County 302058, Taiwan; Department of Anatomy and Cell Biology, National Taiwan University College of Medicine, Taipei 100233, Taiwan; Department of Medical Imaging, National Taiwan University Hospital, Taipei 100225, Taiwan; Department of Neurology, National Taiwan University Hospital, Taipei 100225, Taiwan; Department of Neurology, National Taiwan University Hospital, Taipei 100225, Taiwan; Department of Biomedical Engineering, National Yang Ming Chiao Tung University, Taipei 112304, Taiwan; Department of Neurology, National Taiwan University Hospital, Taipei 100225, Taiwan; Department of Anatomy and Cell Biology, National Taiwan University College of Medicine, Taipei 100233, Taiwan; Graduate Institute of Clinical Medicine, National Taiwan University College of Medicine, Taipei 100229, Taiwan; Center of Precision Medicine, National Taiwan University College of Medicine, Taipei 100025, Taiwan; Department of Medical Imaging, National Taiwan University Hospital, Taipei 100225, Taiwan; Graduate Institute of Medical Device and Imaging, College of Medicine, National Taiwan University Hospital, Taipei 100233, Taiwan; Department of Neurology, National Taiwan University Hospital, Taipei 100225, Taiwan

**Keywords:** amyotrophic lateral sclerosis (ALS), magnetic resonance imaging (MRI), diffusion tensor imaging (DTI), perivascular space, glymphatic system

## Abstract

Perivascular space (PVS) dysfunction may potentially contribute to the development and progression of amyotrophic lateral sclerosis (ALS). This study investigated the clinical relevance of PVS dysfunction in ALS. Two PVS parameters were quantified in patients with ALS: (i) the enlarged perivascular space (ePVS) score and (ii) the diffusion tensor image analysis along the perivascular space (DTI-ALPS) index. These parameters were analysed in relation to the clinical, structural and prognostic features of ALS. The study included 55 patients with ALS (33 men; mean age, 61.38 ± 10.95 years). The DTI-ALPS index was markedly reduced in the patients compared to age- and gender-matched controls, and there were no differences in ePVS scores between the two groups. The ePVS total score was positively correlated with the ALS progression, as measured by the monthly change in the revised ALS functional rating scale. The ePVS basal ganglia regional score was inversely correlated with muscle strength. Additionally, both the ePVS score and the DTI-ALPS index were associated with regional grey matter volumes of the superior frontal gyrus and middle frontal gyrus, and the DTI-ALPS index was associated with diffusion parameters of the corticostriatal and corticothalamic tracts. This study underscores the importance of PVS dysfunction in ALS according to the ePVS and a reduced DTI-ALPS index, which were respectively associated with disease progression, neurological deficits, including reduced muscle strength, and cortical and subcortical structural changes.

## Teaser

Perivascular space dysfunction has been proposed to be a pathogenic mechanism in amyotrophic lateral sclerosis (ALS). By studying MRI-based perivascular space parameters in patients with ALS, Hsueh *et al*. reported that changes and dysfunction in perivascular space imaging parameters may contribute to the progression and clinical manifestations of ALS.

## Graphical Abstract

**Graphical Abstract:**
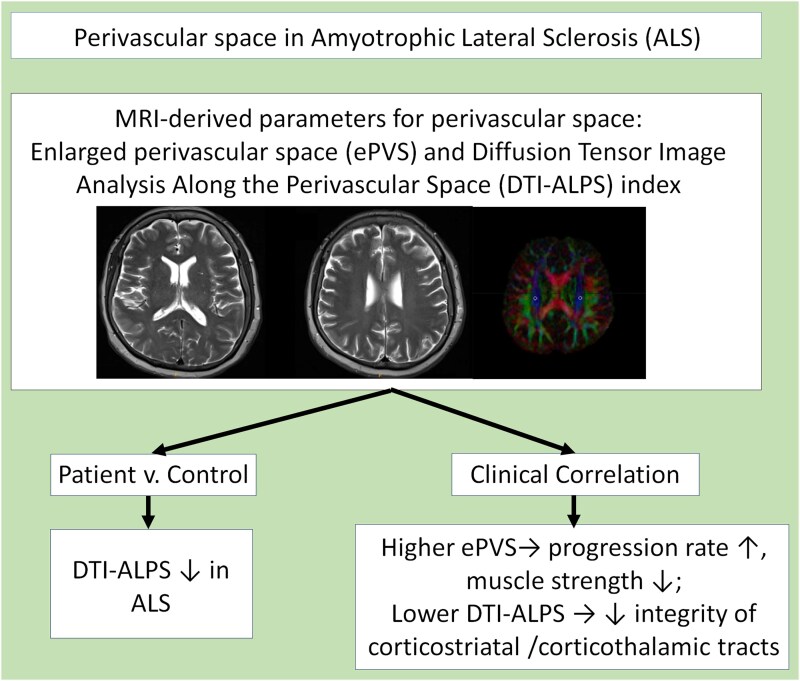

## Introduction

Amyotrophic lateral sclerosis (ALS) is a neurodegenerative disease characterized by the involvement of both upper and lower motor neurons.^1^ It typically manifested with motor symptoms, including muscle weakness, dysphagia, dysarthria and respiratory failure, along with non-motor symptoms, including disinhibition and dementia.^1^ Pathological studies have identified the deposition of TDP-43 in various structures of the central nervous system in patients with ALS following a specific stage-dependent progression pattern, which is recognized as a pathological hallmark of ALS.^2^ Although a small proportion of ALS cases are associated with known genetic mutations,^3^ the aetiology of the majority of cases remains unknown. Several pathophysiological mechanisms have been proposed, including excitotoxicity, oxidative stress, abnormal protein disposal and neuroinflammation.^4^ Given the complexity of these factors, the pathogenesis of ALS is believed to involve a multistep disease process.^5,6^

Neuroimaging studies, particularly those employing magnetic resonance imaging (MRI), have revealed widespread structural changes in cerebral grey and white matter in ALS, with potential clinical implications.^7,8^ However, currently available treatments for ALS are not disease-modifying and can only slightly delay the disease progression. To alter the course of ALS, a deeper understanding of the mechanisms underlying neurodegeneration in the central nervous system is essential. Recent evidence suggests that the dysfunction of perivascular spaces (PVSs) contributes to various neurovascular and neurodegenerative diseases by impairing metabolic clearance in the central nervous system.^9^ This dysfunction may lead to the accumulation of toxic metabolites and abnormal protein disposal,^10,11^ processes implicated in the pathogenesis of ALS. To investigate these mechanisms, many non-invasive neuroimaging techniques have been developed, providing opportunities to explore their roles in neurological disorders.^12^ Two imaging approaches have been widely used in previous studies. The first involves the assessment of enlarged perivascular spaces (ePVSs) by visually grading enlarged T2-hyperintense PVSs in the basal ganglia, centrum semiovale and brainstem on a routine MRI scan based on established criteria.^13^ Previous studies had validated it as a reliable approach for the measurement of PVS dysfunction.^13^ The second approach is the diffusion tensor image analysis along the perivascular space (DTI-ALPS) index,^14^ which evaluates the diffusivity in the centrum semiovale along the deep medullary veins. Although there is still an active debate about the exact underlying structural and pathophysiological basis of the DTI-ALPS index, it has been found to correlate with the findings of intrathecal contrast-mediated imaging, which is considered the gold standard for glymphatic clearance measurement.^15^ Both the ePVS and DTI-ALPS index have been extensively studied and have been demonstrated to be correlated with clinical presentation and progression in various neurological disorders.^12,16^ However, the role of PVS dysfunction in ALS remains poorly understood. Imaging-based assessments of perivascular dysfunction could provide valuable insights into the pathogenesis of ALS. In the present study, we simultaneously assessed the ePVS score and the DTI-ALPS index in patients with ALS, and analysed their correlation with clinical profiles and other structural parameters of brain imaging.

## Materials and methods

### Study design and participants

Patients who were diagnosed with definite or probable ALS based on the revised Awaji Criteria^17^ were enrolled in the study. The participants received evaluation and management at the Department of Neurology of National Taiwan University Hospital, Taipei, Taiwan, between January 2016 and June 2024. Patients were excluded if they had an alternative diagnosis, significant sensory symptoms, other major neurological disorders, intracranial space-occupying lesions or a history of major trauma involving the brain, spinal cord and peripheral nerves. All participants received regular outpatient follow-up, along with individualized medical treatments and supportive care tailored to their clinical needs.

**Figure 1:**
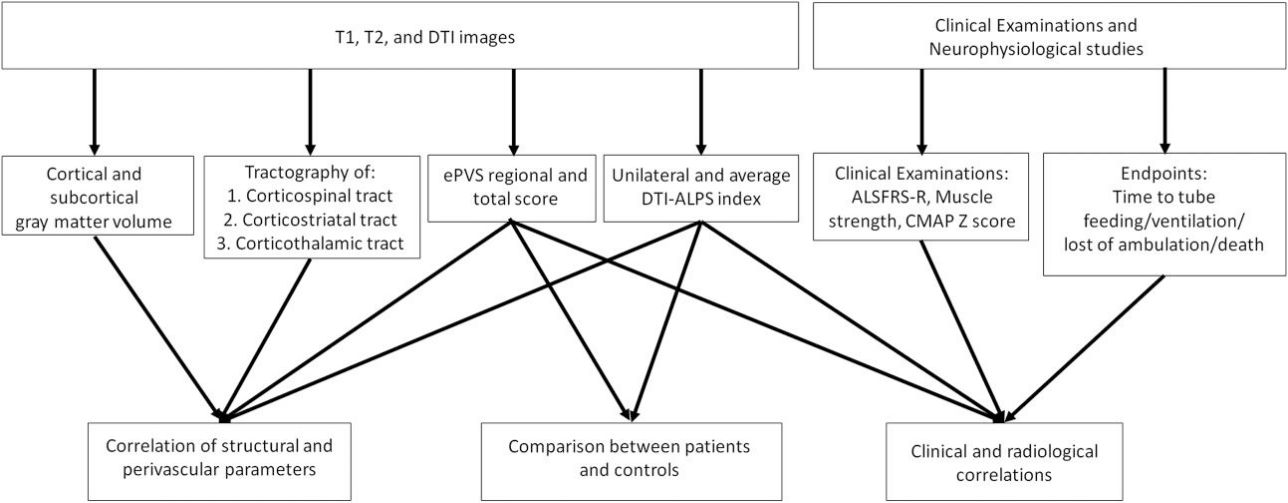
**Overview of study design.** This study investigated the role of PVS dysfunction in ALS by exploring the associations between PVS imaging parameters, clinical profiles and cerebral structural imaging biomarkers. Clinical profiles included (i) the ALSFRS-R, the sum score of muscle strength according to the MRC grading system and the summation of compound muscle action potential *Z* scores (CMAP *Z* sum score) to assess severity of ALS; and (ii) the monthly changes in ALSFRS-R, and time from symptom onset to tube feeding, ventilator dependence, loss of ambulation, or death to evaluate disease progression and prognosis. PVS imaging parameters included the ePVS regional and total scores and the DTI-ALPS index. Cerebral structural imaging biomarkers included tractography of the corticospinal, corticostriatal and corticothalamic tracts and the volume of selected cortical and subcortical grey matter structures.

The enrolled patients underwent standardized clinical examinations and evaluations as described in a previous article.^18^ Global functional deficits were assessed using King’s clinical staging system for ALS (King’s stage)^19^ and the revised ALS functional rating scale (ALSFRS-R).^20^ The average monthly change in ALSFRS-R scores during the first 12 months of follow-up after image acquisition was also calculated separately for each patient by dividing the difference between the baseline and 12-month ALSFRS-R scores by 12. Muscle strength was assessed and quantified using the Medical Research Council (MRC) grading system.^21^ Clinical endpoints included the interval between the symptom onset and the start of nasogastric tube or percutaneous gastrostomy feeding (time-to-tube feeding), the interval between symptom onset and the initiation of mechanical ventilation (time-to-mechanical ventilation), the interval between symptom onset and loss of independent ambulation (time-to-loss of ambulation) and overall survival after symptom onset. The control group was required to meet the following criteria: (i) no neurological symptoms or history of neurological disorders; (ii) no significant structural abnormalities on the neurological images as interpreted by radiologists; (iii) normal findings on neurological examinations and standardized neuropsychiatric tests performed by neurologists; and (iv) no significant renal or hepatic disorders. The study design is presented in Fig. 1.

The study was approved by the Institutional Review Board of National Taiwan University Hospital (Approval number: 202106029RINC and 202401049RINB) and conducted in compliance with the Declaration of Helsinki. Written informed consent was obtained from all participants before all procedures in the study.

### Electrophysiological evaluation

Electromyography and nerve conduction studies were performed for all patients during the initial evaluation using the Nicolet Viking EDX system (Natus Neurology, Middleton, WI, USA) according to the standard protocol for the diagnosis of ALS.^17^ At least two muscles from either the cervical and lumbar segments, along with at least one muscle from either the cranial and thoracic segments, were sampled during electromyography studies, according to established protocols. Sites with significant structural deformity, previous surgical intervention and trauma were avoided during sampling. The motor and sensory nerves of the upper and lower limbs were sampled for nerve conduction studies. Distal compound muscle action potential (CMAP) amplitudes in the median, ulnar, peroneal and tibial nerves on either sides were further standardized by calculating the number of standard deviations from the mean based on the reference values established in our laboratory^22^; this was defined as a *Z* score. The summation of these *Z* scores (CMAP *Z* sum score) was used to estimate the extent of lower motor neuron involvement in ALS.

### Image studies

Brain and cervical MRI was performed using a 3-Tesla whole-body system (Tim Trio, Siemens, Erlangen, Germany) with an eight-channel phased-array head coil and a four-channel phased-array neck coil for signal reception, following our previously reported imaging protocol,^18^ which included three-dimensional T_1_-weighted gradient echo (repetition time (TR) = 2530 ms, echo time (TE) = 2.27 ms and inversion time (TI) = 1100 ms), T_2_-weighted turbo spin echo (TR/TE = 5500/88 ms), fluid-attenuated inversion recovery (TR/TE/TI = 10 000/93/2605 ms) and diffusion tensor imaging (DTI) (TR/TE = 3000/97 ms, *b*-value = 0 and 800 s/mm^2^,^20^ non-collinear directions of diffusion encoding). Note that DTI assumes Gaussian diffusion and has been commonly performed with *b* = 1000 s/mm^2^. We slightly lowered the *b*-value to 800 s/mm^2^ because more recent studies^23,24^ suggest that non-Gaussian diffusion can be present in white matter areas when the *b*-value is as low as 1000 s/mm^2^. The voxel size is 0.25 × 0.25 × 5 mm^3^ for T_2_-weighted images, 0.5 × 0.5 × 5 mm^3^ for FLAIR images, 1 × 1 × 1 for T_1_-weighted images and 2 × 2 × 4 mm^3^ for diffusion images. The slice thickness is 5 mm. We corrected for bulk motion using the Statistical Parametric Mapping software (12th edition) (www.fil.ion.ucl.ac.uk/spm/software/spm12/) and removed corrupted images (e.g. signal dropout and noticeable artefacts) by visual inspection. No further correction was performed for eddy current and distortion. The images were reconstructed using the generalized q-sampling imaging reconstruction method of DSI studio with a diffusion length sampling ratio of 1.25. Tracking and angular thresholds were set at random. The minimal and maximal lengths were 30 and 200 mm, respectively, and the tracking was terminated when 1 000 000 seeds had been placed. The obtained MR images were reviewed by a board-certified neuroradiologist. MR images with significant structural lesions, motion artefacts and possible aetiologies that may mimic ALS, as identified by a board-certified neuroradiologist, were excluded from further analysis.

### Measurement of enlarged perivascular space

**Figure 2:**
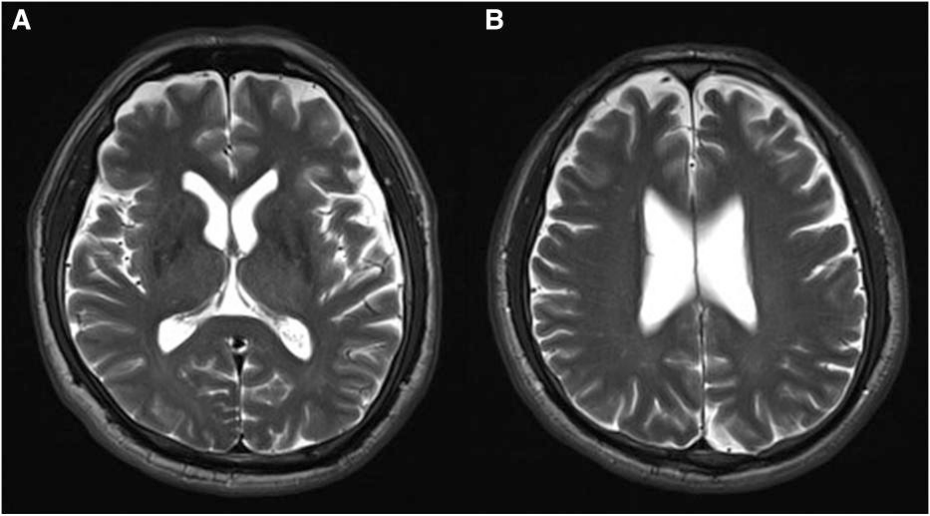
**Example of ePVSs in an ALS patient.** ePVSs located in the basal ganglia, centrum semiovale and midbrain were measured and graded based on the protocol established by Potter *et al*.^13^ The figure is an example of grade 1 ePVS in the basal ganglia (Fig. 2A) and grade 1 ePVS in the centrum semiovale (Fig. 2B).

ePVS was measured following the protocol established by Potter *et al*.^13^ ePVS is defined as small, sharply delineated structures with either a spheroid or linear shape and a diameter of <3 mm. Its signal intensity should be similar to the cerebrospinal fluid on T_2_-weighted imaging, and could be isointense or hypointense on T_1_-weighted imaging. Measurements were performed in the midbrain, the bilateral basal ganglia and the centrum semiovale. At least three slices of images were examined for the basal ganglia and centrum semiovale, while at least two slices of images were examined for the midbrain. For the determination of ePVS in the basal ganglia, the caudate nucleus, thalamus, lentiform nucleus, insular cortex, internal capsule and external capsule were examined and scored. The ePVS count for each location was based on the slice with the highest count. The regional ePVS scores were graded as follows: for the ePVS in the basal ganglia and centrum semiovale, score 0 for absence of ePVS, score 1 for 1–10 ePVS, score 2 for 11–20 ePVS, score 3 for 21–40 ePVS and score 4 for more than 41 ePVS identified; for the ePVS in the midbrain, score 0 if there is absence of ePVS and score 1 if there is presence of ePVS (Fig. 2A and B). The summation of the regional ePVS scores in the bilateral basal ganglia and centrum semiovale, and the midbrain is defined as the ePVS total score.

### Diffusion tensor imaging analysis along the perivascular space index

**Figure 3:**
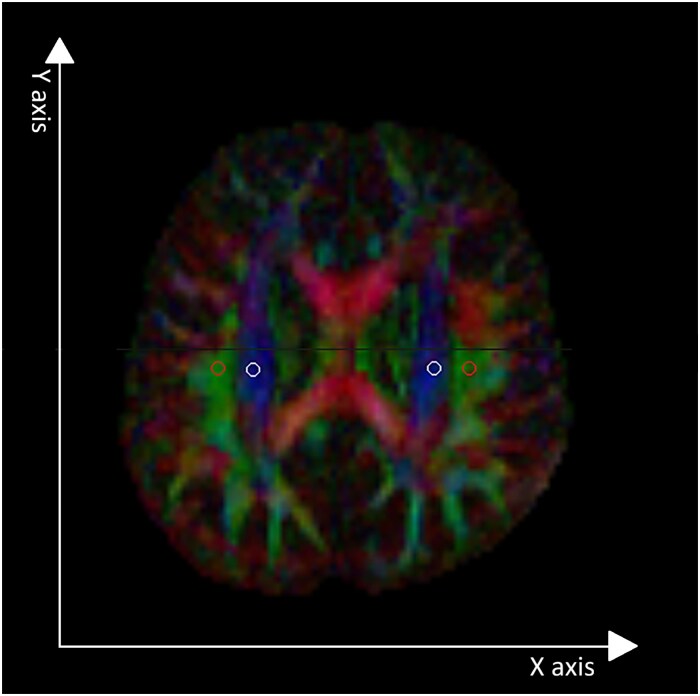
**Quantification of DTI-ALPS.** The DTI colour map of a patient’s brain identified the major fibre projection direction of the white matter fibres (red, *X*-axis projection; green, *Y*-axis projection; blue, *Z*-axis projection). The DTI-ALPS index was calculated by obtaining directional diffusivity of the *X*-axis, *Y*-axis and *Z*-axis from a 5-mm diameter spherical region of interest placed in the projection fibres (white circle) and association fibres (red circle), respectively, at the level around the caudate body and the central part of lateral ventricles using the imaging processing software DSI studio, based on the protocol and formula proposed by Takoa *et al*.^14^

The DTI-ALPS index was calculated following the method proposed by Taoka *et al*.^14^ using the DSI Studio image processing software (http://dsi-studio.labsolver.org).^25^ By referring to diffusion-weighted images and colour maps of fractional anisotropy, the required directional diffusivity of the *X*-axis, *Y*-axis and *Z*-axis was extracted from two spherical regions of interest with a diameter of 5 mm placed within the association fibres and projection fibres, respectively, at the level around the caudate body and the central part of the lateral ventricles. The DTI-ALPS index was calculated separately for each hemisphere, and the bilateral DTI-ALPS indices were also averaged (Fig. 3).

### Preprocessing of grey matter imaging and target regions-of-interest identification

We obtained the grey matter volume from T_1_-weighted three-dimensional images using the software Statistical Parametric Mapping, 12th edition (https://www.fil.ion.ucl.ac.uk/spm/software/spm12/) with the embedded Computational Anatomy Toolbox 12 (http://www.neuro.uni-jena.de/cat).^26^ Also by using the Statistical Parametric Mapping, inter-sequence co-registration within a subject was performed with rigid transformation. The registration between a subject and the group template (such as the regions of interest and masks) was performed with an elastic transformation. Tissue segmentation using the Gaussian mixture model and spatial normalization to the Montreal Neurological Institute coordinates using the DARTEL algorithm were performed.^27,28^ The Neuromorphometrics atlas (http://www.neuromorphometrics.com)^29^ and the CoBrA atlas^30^ were used to define the cortical and subcortical grey matter in the normalized space, respectively. The volume of four cortical (precentral gyrus, superior frontal gyrus, middle frontal gyrus and medial superior frontal gyrus) and two subcortical structures (striatum and thalamus) previously known to correlate with the motor presentation in ALS were calculated. The regional volumes mentioned above were normalized by total intracranial volume (referred to as normalized grey matter volume in the context) in order to reduce variability between subjects.^18^

### Preprocessing of diffusion weighted images for diffusion tensor imaging tractography and estimation of diffusion parameters in the tracts

The Human Connectome Project 842 atlas^31^ was warped to each subject’s native space of DTIs using DSI studio (http://dsi-studio.labsolver.org).^25^ We then identified the corticospinal tract, corticostriatal tract and corticothalamic tract from the images. Fractional anisotropy (FA), mean diffusivity (MD), axial diffusivity (AD) and radial diffusivity (RD) were calculated voxel-wise and separately averaged within each of the tracts.

### Statistical analysis

Numerical variables are presented as means ± standard deviation. Fisher’s exact test was used to evaluate the associations between participant groups and the profile parameters of participants. Student *t*-tests or non-parametric tests were conducted to statistically compare groups based on whether the data followed a Gaussian distribution or not. Single variate regression analysis, along with multivariate regression analysis with the covariance of the model *R*^2^ and standardized correlation coefficients, was used for evaluation of the relationship among image parameters and clinical/structural parameters, with age used as the covariate in multivariate regression analysis. Significance was defined as *P* < 0.05. Corrections for multiple comparisons were not performed. The analyses were performed using STATA13 (StataCorp LLC, College Station, TX, USA).

## Results

### Clinical characteristics of the participants

We enrolled 55 ALS patients (33 males, aged 61.38 ± 10.95 years), all of them meeting the Awaji diagnostic criteria for definite and probable ALS at the time of diagnosis. Forty-five patients had spinal-onset ALS, and 10 had bulbar-onset ALS. Twenty-seven patients had horizontal spreading initially, another 27 had vertical spreading of clinical presentation, and 5 patients had multifocal onset of ALS.

The patients underwent diagnosis assessments at an average of 21.65 ± 21.45 months (range: 5–108 months) after the onset of symptoms. In total, 4, 28 and 19 patients were classified as King’s stage 1, 2 and 3, respectively, at the time of MRI examination, while 4 patients requiring gastrostomy or nasogastric tube feeding were classified as Stage 4 (Table 1). A control group of 37 gender- and age-matched individuals (14 male, *P* = 0.055 compared with the patient group; age 62.95 ± 8.76 years, *P* = 0.474 compared with the patient group) was selected for comparison.

**Table 1:** Clinical characteristics of study participants of patients with ALS and controls

		ALS	Controls	*P*-value
Number		55	37	
Gender (male/female)		33 (60.0)	14 (37.8)	0.055
		22 (40.0)	23 (62.2)	
Age at evaluation (years)		61.38 ± 10.95	62.95 ± 8.76	0.474
Age of onset (years)		59.69 ± 10.64	N/A	
Interval between onset and diagnosis (months)		21.65 ± 21.45	N/A	
King’s stage	1	4 (7.3)	N/A	
	2	28 (51.9)		
	3	19 (34.5)		
	4	4 (7.3)		
Progression pattern	Vertical	27 (49.1)		
	Horizontal	27 (49.1)		
Initial presentation	Focal	50 (90.9)		
	Multiple	5 (9.1)		
Onset site	Spinal	45 (81.8)		
	Bulbar	10 (18.2)		
Metabolic factors	Diabetes mellitus	14 (25.5)	4 (10.8)	0.110
	Hypertension	17 (30.9)	12 (32.4)	1.000
	Hyperlipidemia	16 (29.1)/39	10 (26.3)27	1.000
	Serum Creatine	0.71 ± 0.19	0.81 ± 0.21	

Data are expressed as mean ± SD, or number (proportion).

### Comparison of DTI-ALPS index and ePVS between ALS patients and controls

We first analysed the DTI-ALPS index. Among 46 ALS patients and 28 controls for whom DTI-ALPS index data were available, the average DTI-ALPS index in ALS patients was significantly lower than that in the controls (*P* = 0.005, Cohen’s *D* = 4.197). The PVS parameters for ePVS between ALS patients and the control group were similar: the regional ePVS scores of the basal ganglia (*P* = 0.387), centrum semiovale (*P* = 0.624) or the ePVS total score (*P* = 0.277). There was no association between the total ePVS score and average DTI-ALPS (*P* = 0.802).

### Relationship of perivascular space profiles with clinical profiles

We further analysed the relationship between the clinical profiles of patients and the PVS parameters, using both the ePVS parameters and the average DTI-ALPS index. Neither the ePVS total score nor the DTI-ALPS index was significantly correlated with the patient’s gender, disease duration, disease extent as measured by King’s stage, or clinical progression pattern. The DTI-ALPS index, but not the ePVS total score, had a significant inverse correlation with age (*P* < 0.001, Table 2). No significant correlation was noted between the ePVS total score or average DTI-ALPS index and the presence of vascular risk factors such as diabetes mellitus, hypertension, dyslipidemia or the total number of these vascular risk factors (Supplementary table 1). Regarding the severity of ALS as measured by MRC sum score for limb muscle strength, *Z* sum score of the CMAP amplitude and ALSFRS-R, a significant negative correlation was observed between the regional basal ganglia ePVS score and MRC sum score of contralateral limb muscle strength (supplementary Fig. 1). This relationship remained significant even after age was adjusted for (*P* = 0.030) (Table 3).

**Table 2:** Correlation between ePVS scores and patient characteristics, disease onset patterns, and progression patterns

*β*, *t*, *p*	ePVS total score	DTI-ALPS (average)
Age	−0.000, −0.00, 0.996	−0.0090, −3.90, <0.001* (*R*^2^ = 0.2584)
Gender	0.424, 0.97, 0.334	0.0714, 1.18, 0.246
Disease duration	0.001, 0.07, 0.945	0.0002, 0.17, 0.865
King’s stage	−0.088, −0.30, 0.766	−0.0393, −1.00, 0.325
Vertical/Horizontal	0.481, 1.13, 0.264	0.0999, 1.65, 0.106
Bulbar onset/spinal onset	−0.805, −1.48, 0.145	0.0534, 0.66, 0.510
Unifocal or multifocal	−1.057, −0.93, 0.358	0.1034, 0.96, 0.345

β, beta regression coefficients; *t*, *t* score; *p*, *P* value.

^*^ *P* < 0.05.

**Table 3:** Clinical correlations between regional ePVS scores and the DTI-ALPS index

	Regional ePVS scores			DTI-ALPS index
*β*, *t*, *p*	Basal ganglia	Centrum semiovale	Total score	
Univariate correlation of clinical markers				
Muscle strength (MRC)	−0.023, 2.34, 0.021* (*R*^2^ = 0.0482)	0.003, 0.21, 0.836	−0.015, 0.55, 0.583	0.008, 1.34, 0.185
CMAP *Z* score	−0.009, 0.87, 0.384	−0.000, 0.01, 0.996	0.007, 0.26, 0.797	0.002, 0.32, 0.752
ALSFRS-R	0.001, 0.07, 0.948	−0.017, 0.84, 0.410	−0.022, 0.67, 0.511	0.005, 0.82, 0.425
Multivariate regression of clinical markers (adjusted with age)				
Muscle strength (MRC)	−0.023, 2.20, 0.030* (*R*^2^ = 0.0499)	0.001, 0.08, 0.933	−0.016, 0.56, 0.578	0.004, 0.70, 0.484
CMAP *Z* score	−0.008, 0.74, 0.459	−0.001, 0.12, 0.908	0.007, 0.26, 0.796	−0.003, 0.63, 0.533
ALSFRS-R	0.006, 0.30, 0.769	−0.017, 0.80, 0.430	−0.016, 0.47, 0.643	−0.002, 0.28, 0.783

CMAP, compound muscle action potential; *β*, beta regression coefficients; *t*, *t* score; *p*, *P* value.

^*^ *P* < 0.05.

### Correlation between volumetric and PVS parameters in ALS

We further evaluated the relationship between PVS parameters and cerebral structural parameters, including standardized volume of motor-related grey matter structures such as the precentral gyrus, superior frontal gyrus, middle frontal gyrus, superior medial frontal gyrus, thalamus and striatum. A significant positive correlation was identified between the regional centrum semiovale ePVS scores and the unilateral DTI-ALPS index, with the volume of the ipsilateral superior and middle frontal gyri (Table 4, supplementary Fig. 1).

**Table 4:** Unilateral correlation between grey matter volume and regional ePVS scores

*β*, *t*, *p*	Regional ePVS score			DTI-ALPS index
Structures	Basal ganglia	Centrum semiovale	Total score	
Prefrontal	−70.808, −1.18, 0.240	44.206, 0.50, 0.619	−49.872, −0.13, 0.894	18.504, 0.48, 0.634
Superior medial frontal	−83.089, −1.18, 0.241	150.893, 1.47, 0.147	510.801, 0.88, 0.382	48.127, 1.06, 0.291
Middle frontal	−37.358, −0.89, 0.377	158.246, 2.66, 0.009* (*R*^2^ = 0.0793)	369.148, 1.49, 0.145	72.175, 2.78, 0.007* (*R*^2^ = 0.0863)
Superior frontal	−63.725, −1.05, 0.296	251.293, 2.95, 0.004* (*R*^2^ = 0.0957)	637.161, 1.75, 0.088	117.910, 3.19, 0.002* (R^2^ = 0.1103)
Striatum	22.278, 0.38, 0.703	108.672, 1.28, 0.204	443.547, 1.35, 0.186	63.858, 1.74, 0.086
Thalamus	−0.202, −0.00, 0.998	203.233, 1.31, 0.193	626.168, 0.93, 0.359	45.750, 0.67, 0.504

*β*, beta regression coefficients; *t*, *t* score; *p*, *P* value.

^*^ *P* < 0.05.

### Correlation between diffusion-based and PVS parameters in ALS

The relationship between PVS parameters and diffusion-based parameters, namely, the FA, MD, AD and RD values for the motor-related white matter structures, including the corticospinal, corticothalamic and corticostriatal tracts, was also evaluated. No significant correlation was observed between the ePVS parameters and these diffusion-based parameters. However, higher unilateral ALPS was associated with higher FA, and lower MD, AD and RD of corticostriatal and corticothalamic tracts in patients with ALS (Supplementary Table 2).

We further evaluated the relationship between PVS parameters and cerebral structural parameters. The cerebral structure parameters evaluated included: (i) the standardized volume of motor-related grey matter structures such as the precentral gyrus, superior frontal gyrus, middle frontal gyrus, superior medial frontal gyrus, thalamus and striatum and (ii) the FA, MD, AD and RD values for the motor-related white matter structures, including the corticospinal, corticothalamic and corticostriatal tracts. A significant positive correlation was identified between the regional centrum semiovale ePVS scores and the unilateral DTI-ALPS index, with the volume of the ipsilateral superior and middle frontal gyri (Table 4, Supplementary Fig. 1). No significant correlation was observed between the ePVS parameters and white matter structures. However, higher unilateral ALPS was associated with higher FA, and lower MD, AD and RD of corticostriatal and corticothalamic tracts in patients with ALS (Supplementary Table 2).

### Association between clinical prognosis and PVS parameters

The association between PVS parameters and the clinical prognosis of patients with ALS was evaluated. Prognosis was two metrics: (i) disease progression, measured as the average monthly changes in ALSFRS-R scores within the first year following diagnosis and (ii) time to the fixed endpoints, including the need for tube feeding, loss of ambulation, ventilator dependence and death. Partial correlation analysis revealed that both the ePVS basal ganglia regional and total scores were positively correlated with the monthly decline in ALSFRS-R scores (Table 5). Neither ePVS nor DTI-ALPS demonstrated a significant correlation with the time from disease onset to any of the fixed endpoints.

**Table 5:** Partial single variable correlations between image markers and the rate of decline in ALSFRS-R scores

*β*, *t*, *p*		Standardized monthly changes of ALSFRS-R (S-delta)	S-delta corrected with age
ePVS	ePVS total score	0.522, 2.23, 0.037* (*R*^2^ = 0.1912)	0.545, 2.29, 0.033* (*R*^2^ = 0.2178)
	Regional ePVS scores at basal ganglia	0.300, 2.44, 0.024* (*R*^2^ = 0.2210)	0.310, 2.47, 0.023* (*R*^2^ = 0.2388)
	Regional ePVS scores at centrum semiovale	0.216, 1.25, 0.225	0.217, 1.21, 0.239
Average DTI-ALPS Index		0.044, 1.17 0.261	0.035, 1.08, 0.297

*β*, beta regression coefficients; *t*, *t* score; *p*, *P* value.

^*^ *P* < 0.05.

## Discussion

In this study, we investigated the relationship between the ePVS and the water diffusion pattern along the PVS, quantified by the ePVS score and DTI-ALPS index, respectively. Moreover, their associations with cerebral microstructural characteristics and clinical features in patients with ALS were evaluated. The study demonstrated that (i) the DTI-ALPS index was significantly lower in patients with ALS compared to the controls, while no significant difference in ePVS was observed between the two groups; (ii) higher ePVS scores were associated with a more rapid progression of motor impairment, and elevated regional ePVS scores at basal ganglion were associated with reduced muscle strength; and (iii) a lower DTI-ALPS index was associated with impaired integrity of corticostriatal and corticothalamic tracts, as well as decreased volume of the superior and middle frontal cortices.

PVSs, also known as Virchow–Robin spaces, have been well-characterized in both pathological and radiological studies.^32^ These structures are implied in the clearance of waste and toxic metabolites, either through the glymphatic clearance pathway or the intramural periarterial drainage pathway.^33^ Many non-invasive imaging methods have been applied to evaluate the function of these clearance pathways;^12^ with ePVS and DTI-ALPS being among the most commonly used surrogate parameters. The enlargement of PVSs has been observed in the normal population and in patients with neurological diseases. ePVS hypothesized result from arterial stiffening and protein deposition, which may subsequently impair flow and waste removal in the PVSs.^33^ Pathological studies have linked ePVS with excessive toxic protein accumulation in neurodegenerative diseases, such as Alzheimer’s disease.^34^ Moreover, clinical studies have demonstrated correlations between ePVS scores and the prognosis of vascular and non-vascular neurological disorders.^35,36^ In this study, we documented a strong correlation between ePVS scores and the progression of disease severity in ALS, as determined by the change in ALSFRS-R. This finding suggests PVS dysfunction may contribute to disease progression in ALS, corroborating the concept in other non-vascular neurodegenerative diseases, including Alzheimer’s disease and Parkinson’s disease.^36-38^

The DTI-ALPS index measured the water diffusivity in subcortical white matter regions and has been proposed as a surrogate marker for glymphatic system function.^14^ It had been correlated with findings from intrathecal contrast MR imaging, the gold standard for assessing glymphatic system function.^39^ Previous studies have shown that abnormalities in the DTI-ALPS index could be detected in various neurological disorders and were associated with the progression and clinical presentation of neurodegeneration.^40-42^ Recent studies^43,44^ have indicated a reduced DTI-ALPS index in patients with ALS compared with the general population, suggesting PVS system dysfunction. This observation raised an intriguing question regarding the clinical significance of the DTI-ALPS in ALS. The present study further demonstrated that a reduction in the DTI-ALPS index was significantly associated with lower grey matter volume in the middle and superior frontal cortices, as well as reduced FA in corticothalamic and corticostriatal tracts in patients with ALS, hinting at the potential pathophysiological or therapeutic implications.

In the present study, we examined the relationships between clinical profiles and perivascular parameters. The relationship between muscle strength and ePVS is relatively straightforward: a higher ePVS score, indicative of greater perivascular dysfunction, was associated with reduced muscle power and more rapid progression of clinical impairment. Our findings may suggest that the PVS dysfunction may play as a ‘step’ in the multistep process of ALS, as proposed in previous studies on ALS progression.^5,6^ The changes of PVS may contribute to the development of ALS by interfering with the function of crucial cerebral structures related to motor function, consistent with observations in other neurodegenerative disorders.

Our study also revealed a significant correlation between PVS profiles and cerebral structural changes. Specifically, the DTI-ALPS index was negatively associated with FA in the corticothalamic and corticostriatal tracts, but not in the corticospinal tracts of patients. An interesting feature of our findings is the correlation between regional ePVS and DTI-ALPS with specific regional structural parameters of the brain. Both the ePVS of the centrum semiovale and unilateral DTI-ALPS were positively correlated with the volume of the middle and superior frontal cortices. ALS is increasingly recognized as a multisystem neurodegenerative disease, and regional cerebral neurodegeneration follows an unevenly distributed pattern, which has been demonstrated to be correlated with the deposition of TDP-43.^45^ Certain grey and white matter structures in the brain appear to be more vulnerable to degeneration due to varied levels of susceptibility,^46^ and may be associated with disease progression and clinical manifestations.^18,47^ In the present analysis, the relationships between the regional ePVS score of the centrum semiovale and the unilateral DTI-ALPS index with grey matter volume of the middle and superior frontal cortices were in opposite directions, and there were no associations between the ePVS and DTI-ALPS index. These findings suggest that the ePVS and DTI-ALPS index likely reflect different functional or structural domains of the PVS. Furthermore, it is important to acknowledge the existing criticisms about the accuracy and limitations of the DTI-ALPS method, including questions about its true reflection of glymphatic flow, its dependency on positioning during imaging acquisition, the assumption about the anatomical relationship between glymphatic flow and blood vessels, and the lack of concrete correlation with pathological and imaging studies.^48^ It is possible that the DTI-ALPS index may, in fact, indicate other structural changes in the brain, such as degeneration of the white matter. Further investigation is warranted to elucidate the underlying pathophysiological mechanisms reflected in these imaging findings, particularly in the context of differential brain degeneration in ALS. The pathophysiological mechanism behind these image findings, which might contribute to the differential degeneration of the brain in ALS, warrants further investigation through contrast-based MRI techniques in research.^49^

This study has several limitations. First, there was a significant difference in sex distribution between the patient and control groups due to the restricted funding and instrumental access, and the limited number of scientifically eligible participants in a single-centre study. Nevertheless, no significant differences were observed when the effect of gender was considered in regression analysis, and the calculated *post hoc* power is 100%. Second, although our findings suggest that changes in ePVS and DTI-ALPS may play a role in the development and progression of ALS, they do not establish a direct pathogenic relationship. The DTI-ALPS index and DTI parameters were both derived from DTI sequences, and thus their correlation should be interpreted with caution and requires further investigation.^50^ Third, as a cross-sectional observational study, evaluating the temporal changes of ePVS and DTI-ALPS in the patients and correlating them with the clinical progression was challenging. Fourth, this study applied ePVS scores and DTI-ALPS indexes as surrogate parameters for glymphatic function. Besides these two approaches, glymphatic function in humans could also be evaluated using other imaging approaches, including contrast-enhanced MRI, arterial spin imaging, phase contrast MRI, and other structural parameters such as choroid plexus volume.^49^ Further incorporation and comparison of different approaches may give us a more complete picture of the glymphatic system in humans. Fifth, since corrections for multiple comparisons were not employed, there remains a possibility that some results may be false positives.

In summary, our study revealed correlations between PVS parameters and various aspects of ALS, including structural, clinical and prognostic features. These findings suggest that PVS dysfunction may contribute to the pathogenesis and development of ALS. Further investigation is warranted to understand the exact mechanism under these findings.

## Supplementary Material

fcaf448_Supplementary_Data

## Data Availability

The data and analysis code are available from the corresponding author upon reasonable request following approval by the Institutional Review Board and in compliance with patient privacy protection.
