# Supplementary material for: Implications of perivascular spaces in amyotrophic lateral sclerosis: clinical significance and structural correlation
Source: Brain Commun. 2025 Nov 12;7(6):fcaf448. doi: 10.1093/braincomms/fcaf448 (PMC12645842; doi:10.1093/braincomms/fcaf448)
Supplement: fcaf448_Supplementary_Data [file fcaf448_supplementary_data.docx]

Supplementary Figure 1. Significant correlation of perivascular space parameters with clinical and structural parameters

| Correlation of perivascular space parameters with muscle strength | |
| --- | --- |
|  CI of slope -3.807 to -0.3114, F= 5.464, p=0.0213, R^2^= 0.04816 | |
| Correlation of perivascular space parameters with gray matter structural parameters | |
|   CI of slope 0.0001253 to 0.0008767, F= 7.061, p= 0.0095, R^2^= 0.07928 |   CI of slope 0.0003395 to 0.002052, F=7.744, p= 0.0067, R^2^= 0.08629 |
|   CI of slope 0.0001233 to 0.0006386, F= 8.682, p=0.0042 R^2^= 0.09574 |   CI of slope 0.0003507 to 0.001520, F = 10.16, p= 0.0020, R^2^= 0.1103 |

Abbreviations: CI, confidence interval; ePVS, enlarged perivascular space; DTI-ALPS, diffusion tensor image analysis along the perivascular space; MRC, medical research council; F, F score; p, P score.

Supplementary Table 1: Correlation between perivascular parameters and vascular risk factors

| Risk factors | Diabetes mellitus | Hypertension | Hyperlipidemia | Number of vascular risk factors |
| --- | --- | --- | --- | --- |
| ePVS total score | -0.551, -1.13, 0.265 | 0.026, 0.06, 0.955 | -0.239, -0.51, 0.616 | -0.154, -0.71, 0.484 |
| Average DTI-ALPS Index | -0.120, -0.36, 0.722 | -0.322, -0.95, 0.347 | -0.282, -0.83, 0.410 | -0.724, -0.98, 0.333 |

Abbreviations:

DTI-ALPS, diffusion tensor imaging along perivascular space; ePVS, enlarged perivascular space.

Supplementary Table 2. Correlation between the unilateral DTI-ALPS index and diffusion metrics in with white matter tracts

| β, t, p | | Unilateral DTI-ALPS Index |
| --- | --- | --- |
| Corticospinal tract | FA | 0.624, 0.48, 0.634 |
|  | MD | -0.529, -1.30, 0.198 |
|  | AD | -0.595, -1.56, 0.122 |
|  | RD | -0.457, -1.12, 0.267 |
| Corticostriatal tract | FA | 4.928, 3.53, 0.001*, (R^2^=0.1219) |
|  | MD | -1.155, -3.99, <0.001*, (R^2^=0.1502) |
|  | AD | -1.089, -3.81, <0.001*, (R^2^=0.1387) |
|  | RD | -1.174, -4.05, <0.001*, (R^2^=0.1541) |
| Corticothalamic tract | FA | 3.599, 2.78, 0.007*, (R^2^=0.0792) |
|  | MD | -1.152, -4.48, <0.001*, (R^2^=0.1824) |
|  | AD | -1.167, -4.64, <0.001*, (R^2^=0.1929) |
|  | RD | -1.132, -4.37, <0.001*, (R^2^=0.1753) |

Abbreviations:

DTI-ALPS, diffusion tensor imaging along perivascular space; FA, fractional anisotropy, MD, mean diffusivity; AD, axial diffusivity; RD, radial diffusivity.

*: p<0.05
